# Formaldehyd

**Published:** 1897-07

**Authors:** 


Formaldehyd is at present the most popular antiseptic and disinfectant. It is usually found in the market as a forty-percent solution. Eight to ten percent destroys the spores of micro-organisms in ten minutes. A one-percent solution destroys cultures within an hour, and disinfects and renders fæces odorless. A three-percent solution will remove all infection from the hands, and one part in ten thousand prevents the growth of pathogenic micro-organisms. Our German, English, French, Spanish and American exchanges are sounding its praises.

---

In Wisconsin any one having a diploma from an incorporated medical college may practic as a physician, and any three persons may incorporate themselves as a medical college and issue diplomas, without any instruction, and the diplomas cannot be questioned. To improve on this state of affairs a bill is to be presented to the Wisconsin Legislature.—*Am. Medico-Surg. Bulletin.*

---

The first fine for spitting in the cars was imposed upon a man in this city who violated the health ordinance. The amount of fine was $5.00. The man pleaded ignorance of the law, but without avail. A few such lessons to passengers in the elevated and other cars, particularly of the east side, would prove to be wholesome reminders of ordinary decency.—*N. Y. Med. Record.*

---

During the administration of ether the most alarming danger signals are sudden pallor of the face, dilatation of the pupils, and darkening of the blood. When these symptons present themselves the anesthetic should at once be withdrawn and resuscitating measurers instituted.—*Hearn.*

